# Simultaneous Carriage of *mcr-1* and Other Antimicrobial Resistance Determinants in *Escherichia coli* From Poultry

**DOI:** 10.3389/fmicb.2018.01679

**Published:** 2018-07-25

**Authors:** Johana E. Dominguez, Leandro M. Redondo, Roque A. Figueroa Espinosa, Daniela Cejas, Gabriel O. Gutkind, Pablo A. Chacana, José A. Di Conza, Mariano E. Fernández Miyakawa

**Affiliations:** ^1^Laboratorio de Bacteriología General, Instituto de Patobiología, Centro Nacional de Investigaciones Agropecuarias, Instituto Nacional de Tecnología Agropecuaria, Buenos Aires, Argentina; ^2^Consejo Nacional de Investigaciones Científicas y Tecnológicas, Buenos Aires, Argentina; ^3^Laboratorio de Resistencia Bacteriana, Cátedra de Microbiología, Universidad de Buenos Aires, Facultad de Farmacia y Bioquímica, Buenos Aires, Argentina

**Keywords:** Colistin, *mcr-1*, food-borne bacteria, *Escherichia coli*, CTX-M-2, *qnrB*, multi drug resistance

## Abstract

The use of antimicrobial growth promoters (AGPs) in sub-therapeutic doses for long periods promotes the selection of resistant microorganisms and the subsequent risk of spreading this resistance to the human population and the environment. Global concern about antimicrobial resistance development and transference of resistance genes from animal to human has been rising. The goal of our research was to evaluate the susceptibility pattern to different classes of antimicrobials of colistin-resistant *Escherichia coli* from poultry production systems that use AGPs, and characterize the resistance determinants associated to transferable platforms. *E. coli* strains (*n* = 41) were obtained from fecal samples collected from typical Argentine commercial broiler farms and susceptibility for 23 antimicrobials, relevant for human or veterinary medicine, was determined. Isolates were tested by PCR for the presence of *mcr-1*, extended spectrum β-lactamase encoding genes and plasmid-mediated quinolone resistance (PMQR) coding genes. Conjugation and susceptibility patterns of the transconjugant studies were performed. ERIC-PCR and REP-PCR analysis showed a high diversity of the isolates. Resistance to several antimicrobials was determined and all colistin-resistant isolates harbored the *mcr-1* gene. CTX-M-2 cefotaximase was the main mechanism responsible for third generation cephalosporins resistance, and PMQR determinants were also identified. In addition, co-transference of the *qnrB* determinant on the *mcr-1*-positive transconjugants was corroborated, which suggests that these resistance genes are likely to be located in the same plasmid. In this work a wide range of antimicrobial resistance mechanisms were identified in *E. coli* strains isolated from the environment of healthy chickens highlighting the risk of antimicrobial abuse/misuse in animals under intensive production systems and its consequences for public health.

## Introduction

Antimicrobial agents have been used extensively for prevention and treatment of infectious diseases in food animals (Dibner and Richards, 2005; Niewold, 2007). The concomitant risk of spreading antibiotic resistance to human population through the food supply chain and the environment is important since many classes of these antimicrobial agents are also used in human medicine. Therefore, increased global concern regarding development of antimicrobial resistance and transference of resistance genes from animals to humans has been rising (Ljungquist et al., 2016; Madec et al., 2017; Wang et al., 2017).

Various antimicrobials have been widely used by the poultry industry as antibiotic growth promoters (AGPs) since the 1950s. To reduce costs of production, AGPs have been added into feed to promote weight gain by optimizing feed conversion ratios (Moore and Evenson, 1946; Jukes et al., 1950). In contrast to therapeutic usages of antimicrobials that are administered at high doses for a limited period of time, AGPs are used in sub-therapeutic doses during longer periods. This situation is particularly favorable for the selection of resistant microorganisms (Diarra et al., 2007).

Any use of antimicrobial agents may contribute to clinical relevant antimicrobial resistance. One of the first findings that led to strong recommendations (and even banning) for the use of AGP in the European Union (EU) was the finding that administration of avoparcin, a glycopeptide AGP, was involved in emerging glycopeptide-resistant bacteria (Howarth and Poulter, 1996). In the same way, use of colistin as an AGP in livestock led to the emergence and silent dissemination of plasmid-mediated mechanisms involved with polymyxin resistance (Rhouma et al., 2016). International organizations responsible for human, animal health, and food production (World Health Organization-WHO/World Organization for Animal Health-OIE/ Food and Agriculture Organization-FAO) carried out systematic evaluations on the impact of veterinary antimicrobial resistance on public health, and they stated that the misuse and overuse of antimicrobials is accelerating the processes of antimicrobial resistance. As a result, this topic is now considered as one of the critical issues in developed and developing countries as indicated by the United Nations General Assembly in 2016.

As part of a technical support program to national poultry producers, our team conducted studies to understand the antimicrobial resistance evolution in food-borne bacteria under commercial production systems in Argentina. Our studies included the selection of *Escherichia coli* as an indicator microorganism and concluded that almost 50% of the strains were found to be resistant to colistin used as AGP (Dominguez et al., 2017) which was much higher than reported in studies published previously. Therefore, the aim of this work was to evaluate the susceptibility pattern to different classes of antimicrobials of colistin-resistant *E. coli* isolated from poultry production systems that use AGP, and to characterize the resistance determinants associated to transmissible elements.

## Materials and methods

### Sampling and *E. coli* isolation

Fresh fecal samples were collected from 129 commercial broiler farms located in the most relevant production areas of Argentina (Entre Rios and Buenos Aires Provinces). At the moment of the sampling, healthy 4–6 week-old broiler chickens were at the end of the rearing cycle in the farms (Dominguez et al., 2017). Each *E. coli* strain was isolated from a pool of 10 feces samples collected in different sections of each barn. All samples were placed into boxes containing ice packs and immediately transported to the laboratory to isolate the microorganism by culture on non-antibiotic-supplemented MacConkey agar plates at 37°C for 18–24 h. Isolates were initially selected by the morphology of the colonies and further identified by standard biochemical tests (Brenner and Farmer, 2015). According to the size of the farms, a fixed number of isolates were arbitrarily selected: 2 isolates from small (less than 50,000 birds), 3 from medium (between 50,000 and 150,000 birds) and 6 from large (more than 150,000 birds) farms. Overall 304 *E. coli* isolates were obtained (Dominguez et al., 2017). In the present study a subset of 31 strains resistant to colistin and 10 susceptible -according to EUCAST criteria- were analyzed (EUCAST 2017)^1^. These strains were isolated from 11 farms belonging to 3 different integrated companies located at Entre Rios and Buenos Aires Provinces.

### Phenotypic antimicrobial susceptibility testing

Antibiotic susceptibility was determined by agar disk diffusion test against 23 antibiotics representing seven antimicrobial classes, commonly used in human and veterinary medicine. Antimicrobial susceptibility was determined for the following agents:

- ß-lactams including:
  ■ Penicillins: Ampicillin (AMP), Amoxicillin-Clavulanic Acid (AMC)
  ■ Second generation cephalosporins: Cefuroxime (CXM)
  ■ Third generation cephalosporins (TGC): Ceftiofur (CFT), Cefotaxime (CTX), Ceftriaxone (CRO), Ceftazidime (CAZ)
  ■ Cephamycins: Cefoxitin (FOX)
  ■ Fourth generation cephalosporins: Cefepime (FEP)
  ■ Monobactams: Aztreonam (ATM)
  ■ Carbapenems: Imipenem (IMI), Meropenem (MEM)
- Aminoglycosides: Kanamycin (KAN), Gentamicin (GEN), Amikacin (AMI), Streptomycin (STR)
- Tetracyclines: Tetracycline (TET)
- Quinolones: Nalidixic Acid (NAL), Ciprofloxacin (CIP), Enrofloxacin (ENR)
- Sulfonamides: Trimethoprim-Sulfamethoxazole (SXT)
- Phenicols: Chloramphenicol (CLR)
- Polymyxins: Colistin (COL)

The results were interpreted according to the Clinical and Laboratory Standards Institute (CLSI) criteria, (CLSI, 2017) and (CLSI, 2013). Susceptibility to colistin was evaluated by broth microdilution and results were interpreted according to the European Committee on Antimicrobial Susceptibility Testing guidelines (EUCAST).

*E. coli* strains resistant to three or more antimicrobial classes were categorized as multidrug resistant (MDR). Phenotypic screening for extended spectrum β-lactamase (ESBL) and plasmid mediated AmpC (pAmpC) was conducted performing synergy test using cefotaxime/clavulanic acid (CTX/CLA, 30/10 μg), ceftazidime/clavulanic acid (CAZ/CLA, 30/10 μg) and phenyl-boronic acid (PBA, 300 μg) containing disks, respectively (Yagi et al., 2005; CLSI, 2017). *E. coli* ATCC 25922 and *E. coli* ATCC 35218 were included as control.

### Molecular analysis of resistance

All strains were tested by PCR for the presence of transferable resistance markers (*mcr-1*, ESBL, *pAmpC*, and plasmid mediated quinolone resistance—PMQR- coding- genes) using primers listed in Table 1. In the case of *mcr-1* detection, the full *mcr-1* gene was amplified and sequenced by using CLR5-F in combination with MCR1-R (5′-TGCGGTCTTTGACTTTGTC) (this study). Total DNA was obtained by boiling bacterial suspensions and plasmid DNA was purified according to Kado and Liu method (Kado and Liu, 1981).

**Table 1:** Targets, primers, sequence, and product size used for PCR and sequencing of *mcr-1*, BLEE, ESBL, AmpC, and PMQR genes.

**Targets**	**Primers**	**Nucleotide Secuence (5′-3′)**	**Size (bp)**	**References**
*mcr-1*	CLR5-F	CGGTCAGTCCGTTTGTTC	344	Liu et al., 2016
	CLR5-R	CTTGGTCGGTCTGTA GGG		
*bla*_CTX−M−like_	CTX-M GRAL F	ATGTGCAGYACCAGTAARGTKATGGC	500	Ghiglione, 2015
	CTX-M GRAL R	CCGCTGCCGCTYTTATCVCCBAC		
*bla*_CTX−M−group1_	CTX-M-1 CF	ATGGTTAAAAAATCACTGC	864	Saba Villarroel et al., 2017
	CTX-M-1 CR	GGTGACGATTTTAGCCGC		
	CTX-M-1 FpK	AAATGGTTAAAAAATCACTGC	876	Ghiglione, 2015
	CTX-M-1 RpK	CTACAAACCGTCGGTGACGAT		
*bla*_CTX−M−group2_	CTX-M-2 FpK	TAATGATGACTCAGAGCATTCGC	900	Ghiglione, 2015
	CTX-M-2 RpK	GCATCAGAAACCGTGGGTTACG		
	CTX-M-2 CF	TTAATGATGACTCAGAGCATTC	910	Bertona et al., 2005
	CTX-M-2 CR	GATACCTCGCTCCATTTATTGC		
*bla*_CTX−M−group8_	CTX-M-8 CF	TGAATACTTCAGCCACACG	923	Saba Villarroel et al., 2017
	CTX-M-8 CR	TAGAATTAATAACCGTCGGT		
	CTX-M-8 FpK	AGATGATGAGACATCGCGTTAAGC	1184	Ghiglione, 2015
	CTX-M-8 RpK	TTAATAACCGTCGGTGACG		
*bla*_CTX−M−group9_	CTX-M-9 CF	ATGGTGACAAAGAGAGTGC	876	Saba Villarroel et al., 2017
	CTX-M-9C R	TCACAGCCCTTCGGCGATG		
	CTX-M-9 FpK	AGATGGTGACAAAGAGAGTGC	876	Ghiglione, 2015
	CTX-M-9 RpK	TTACAGCCCTTCGGCGATG		
*bla*_CTX−M−group25_	CTX-M-25 CF	ATGAGAMAWMGCGTWARGC	878	Saba Villarroel et al., 2017
	CTX-M-25 CR	TAGAATTAATAACCGTCGGTGAC		
*bla*_pAmpC_	MOXMF	GCT GCT CAA GGA GCA CAG GAT	520	Cejas et al., 2012
	MOXMR	CAC ATT GAC ATA GGT GTG GTG C		
	CITMF	TGG CCA GAA CTG ACA GGC AAA	462	
	CITMR	TTT CTC CTG AAC GTC GCT GGC		
	DHAMF	AAC TTT CAC AGC TGT GCT GGG T	405	
	DHAMR	CCG TAC GCA TAC TGG CTT TGC		
	ACCMF	AAC AGC CTC AGC AGC CGG TTA	346	
	ACCMR	TTC GCC GCA ATC ATC CCT AGC		
	EBCMF	TCG GTA AAG CCG ATG TTG CGG	302	
	EBCMR	CTT CCA CTG CGG CTG CCA GTT		
	FOXMF	AAC ATG GGG TAT CAG GGA GAT G	190	
	FOXMR	CAA AGC GCG TAA CCG GAT TGG		
*bla*_CMY−2_	CMY -F	ATGATGAAAAAATCGTTATGCT	1146	
	CMY-R	TTATTGCAGCTTTTCAAGAATGCG		
*qnrA*	qnrA-F	AGAGGATTTCTCACGCCAGG	580	Cruz et al., 2013
	qnrA-R	TGCCAGGCACAGATCTTGAC		
*qnrS*	qnrS-F	GCAAGTTCATTGAACAGGGT	428	
	qnrS-R	TCTAAACCGTCGAGTTCGGCG		
*qnrC*	qnrC-F	GGGTTGTACATTTATTGAATCG	330	
	qnrC-R	CACCTACCCATTTATTTTCA		
*qnrD*	qnrD-F	CGAGATCAATTTACGGGGAATA	582	
	qnrD-R	AACAAGCTGAAGCGCCTG		
*qnrB*	qnrB-F	GGMATHGAAAATCGCCACTG	264	
	qnrB-R	TTTGCYGYYCGCCAGTCGAA		
	qnrBIF-F	ATGWYGYCATTACTGTATA	676	
	qnrBIF-R	CCMATHAYMGCGATRCCAAG		
	qnrBcf-F	GTTRGCGAAAAAATTRACAG	626	
	qnrBIF-R	CCMATHAYMGCGATRCCAAG		
*qepA*	qepA-F	ACATCTACGGCTTCTTCGTCG	501	
	qepA-R	AACTGCTTGAGCCCGTAGATC		
*acc(6′)-lb*	aac(6′)Ib-F	CGATCTCATATCGTCGAGTGTT	447	
	aac(6′)Ib-R	TTAGGCATCACTGCGTGTTC		
*oqxA*	oqxA-F	CTCGGCGCGATGATGCT	393	
	oqxA-R	CCACTCTTCACGGGAGACGA		
*oqxB*	oqxB-F	TTCTCCCCCGGCGGGAAGTAC	513	
	oqxB-R	CTCGGCCATTTTGGCGCGTA		

### Plasmid conjugation studies

To assess *mcr-1* plasmid transferability, conjugation studies by liquid mating were performed*. Salmonella* M1744 and *E. coli* J53 strains were used as recipient and randomly chosen *mcr-1*-positive strains from each farm were used as donors. After the conjugation, the transconjugants obtained from *Salmonella* M1744 were selected in TSA media supplemented with colistin (2 μg/mL), whereas those obtained from *E. coli* J53 were selected with sodium azide (200 μg/mL) and colistin (1 μg/mL). To confirm successful conjugation, colonies obtained in the selective media were screened for *mcr-1* gene by PCR and then colistin MIC was determined for both transconjugant and parental *E. coli* strains by the broth microdilution as described before. In addition, co-resistance to other antimicrobials was assessed by agar disk diffusion method as previously described.

### Molecular typing by PCR-based techniques

Clonality of the isolates was determined by the homology relationships among fragments amplified by ERIC-PCR (Enterobacterial Repetitive Intergenic Consensus) and REP-PCR (Repetitive Extragenic Palindromic) according to Versalovic et al. (1991). Dendrograms were constructed by GelJ 1.0 program, using UPGMA algorithm and applying the DICE correlation coefficient.

### Statistical analysis

Significant differences (*p* < 0.05) in the association among strains according to the presence of genes were determined by Pearson's Chi-squared test with Yates continuity correction using Epidat software (version 4.1).

## Results and discussion

### Resistance to colistin and *mcr*−1 gene detection

The presence of *mcr-1* in Argentina was already detected in *E. coli* isolates recovered from invasive infections in humans (Rapoport et al., 2016) and has also been found in bacteria isolated from domestic animals (Dominguez et al., 2017). The *E. coli* strains included in the present report were classified in the base of their susceptibility to colistin following the recommendations of the European Committee on Antimicrobial Susceptibility Testing (EUCAST, 2017). All strains considered resistant to colistin harbored the *mcr-1* gene as demonstrated by PCR, and the sequenced gene was identical to the previously published sequence, accession number KP347127.1 (Liu et al., 2016). Additionally, from the 10 strains classified as colistin-susceptible, 3 of them were positive for the *mcr-1* gene (Table 2).

**Table 2:** Characteristics of *Escherichia coli* recovered from different farms in Buenos Aires and Entre Ríos, Argentina, 2014.

**Provinces**	**Farms**	**Strain**	**MIC to colistin (μg/mL)**	Resistance determinant^*^								
				***mcr-1***	***AmpC***	***bla*_CTX-M_**	***qnrA***	***qnrB***	***qnrD***	***qnrS***	***oqxAB***	***qepA***
Buenos Aires	1	*E. coli* 190-02	2 (S)									
		*E. coli* 241-S1	8 (R)			**CTX-M-14**						
		*E. coli* 241-S3	1 (S)									
	2	*E. coli* 190-06	0.5 (S)									
		*E. coli* 241-S2	1 (S)									
		*E. coli* 241-S4	1 (S)			**CTX-M-2**						
	3	*E. coli* 241-L1	8 (R)									
		*E. coli* 241-L2	2 (S)			**CTX-M-2**						
		*E. coli* 190-08	8 (R)		**CMY-2**	**CTX-M-2**						
		*E. coli* 190-10	8 (R)			**CTX-M-2**+ **CTX-M14**						
		*E. coli* 241-L3	0,5 (S)			**CTX-M-2**						
	4	*E. coli* 190-13	8 (R)			**CTX-M-2**+ **CTX-M14**						
		*E. coli* 190-14	8 (R)			**CTX-M-2**						
		*E. coli* 241-P2	4 (R)			**CTX-M-2**						
		*E. coli* 241-P3	8 (R)		**CMY-2**							
		*E. coli* 241-P4	2 (S)			**CTX-M-2**+ **CTX-M14**						
		*E. coli* 241-P1	8(R)			**CTX-M-2**						
	5	*E. coli* 190-15	8(R)									
		*E. coli* 190-16	4 (R)									
		*E. coli* 241-Z1	8 (R)			**CTX-M-2**						
		*E. coli* 241-Z2	16 (R)			**CTX-M-2**						
		*E. coli* 241-Z3	8(R)			**CTX-M-2**						
	6	*E. coli* 190-17	8 (R)		**CMY-2**	**CTX-M-2**						
		*E. coli* 190-18	4 (R)			**CTX-M-2**						
		*E. coli* 241-K1	8 (R)			**CTX-M-14**						
		*E. coli* 241-K2	8 (R)			**CTX-M-2**						
		*E. coli* 241-K3	2 (S)			**CTX-M-2**						
		*E. coli* 241-K4	8 (R)			**CTX-M-2**+ **CTX-M14**						
	7	*E. coli* 241-B2	2 (S)									
		*E. coli* 241-B3	8 (R)			**CTX-M-2**						
		*E. coli* 241-B1	4(R)			**CTX-M-2**						
Entre Ríos	8	*E. coli* 191-08	8 (R)			**CTX-M-2**						
		*E. coli* 191-07	8 (R)		**CMY-2**	**CTX-M-2**						
	9	*E. coli* 191-11	8 (R)									
		*E. coli* 191-12	32 (R)									
		*E. coli* 191-13	16 (R)									
	10	*E. coli* 191-16	4 (R)									
		*E. coli* 191-17	8 (R)			**CTX-M-2**						
	11	*E. coli* 191-21	8 (R)			**CTX-M-2**						
		*E. coli* 191-23	32 (R)			**CTX-M-2**+ **CTX-M14**						
		*E. coli* 191-22	8 (R)			**CTX-M-2**						

* *The squares in gray indicate presence of the gene; while the squares in white indicate absence of the studied gene. (R) resistant and (S) susceptible by MIC determinations with colistin*.

From the 31 colistin resistant/*mcr-1*-positive *E. coli* strains, 28 showed MICs ranging from 4 to ≥ 32 μg/mL. Although the disk diffusion method (10 μg colistin disk) is not yet standardized for polymyxins, all 28 strains displayed colistin inhibition zone ≤11 mm. The remaining strains (3/31) showed MICs between 4 and 8 μg/mL but nevertheless displayed inhibition zones ≥ 11 mm with colistin. Although molecular detection is the most appropriate technique for *mcr-1* identification, a strong association with the phenotypic methodologies to detect *mcr-1*-mediated colistin resistance was observed.

Previous works describe the transferable nature of the *mcr-1* gene (Liu et al., 2016). In the present work, the *mcr-1*-mediated colistin resistance was successfully transferred by conjugation to both recipient laboratory strains (*E. coli* J53 and *Salmonella* M1744). Two out of ten *mcr-1*-positive strains from each farm (Table 3) were obtained from liquid mating experiments performed using poultry *E. coli* strains as plasmid donors. Plasmids carrying the *mcr-1* gene conjugated at a transfer frequency of ~1.5 × 10^−3^ transconjugants per donor cell. Accordant to results obtained by Liu et al. (2016), we found that MIC for colistin of the transconjugants increased four- and eight-fold compared to the original recipient strains.

**Table 3:** Plasmid conjugation studies.

		Disk diffusion test^*^		**PCR**
**Strains**	**MIC to colistin (μg/mL)**	**NAL**	**CIP**	***mcr-1*/PMQR/CTX-M**
*E. coli* 190-14	8	R	R	*mcr-1+ qnrB+qnrS*+CTX-M-2
EC190-14 TC^**^	4	I	S	*mcr-1+ qnrB*
*E. coli* 191-07	8	I	S	*mcr-1+ qnrB*+ CTX-M-2+ CMY-2
EC191-07 TC^**^	4	I	S	*mcr-1+ qnrB*
*E. coli* J53	0,5	S	S	–
EC191-07 TCS^***^	8	I	S	*mcr-1+ qnrB*
*Salmonella* M1744	1	S	–	–

* (R) resistant, (I) intermediate and (S) susceptible by disk diffusion test: Nalidixic Acid (NAL), Ciprofloxacin (CIP).

** TC: transconjugants obtained using E. coli J53 as the recipient strain.

*** *TCS: transconjugants obtained using Salmonella M1744 as the recipient strain*.

Molecular typing analysis by the ERIC-PCR and REP-PCR showed that all *E. coli* carrying *mcr-1* gene from this study had high clonal diversity and thus considered as genetically unrelated Figure S1. These results are in concordance with previous reports that describe the wide distribution of the *mcr-1* gene among *E. coli* isolates independently of bacterial source or host species, suggesting a non-clonal spread of colistin resistance (Fernandes et al., 2016; Rapoport et al., 2016). In addition, this study reports a successful plasmid–gene combination of these *E. coli* strains in healthy broiler chickens, which may play a role in the emergence and spread of this gene.

### Resistance to other antimicrobials

#### Resistance to fluoroquinolones and detection of plasmid-mediated quinolone resistant (PMQR) genes

**Figure 1:**
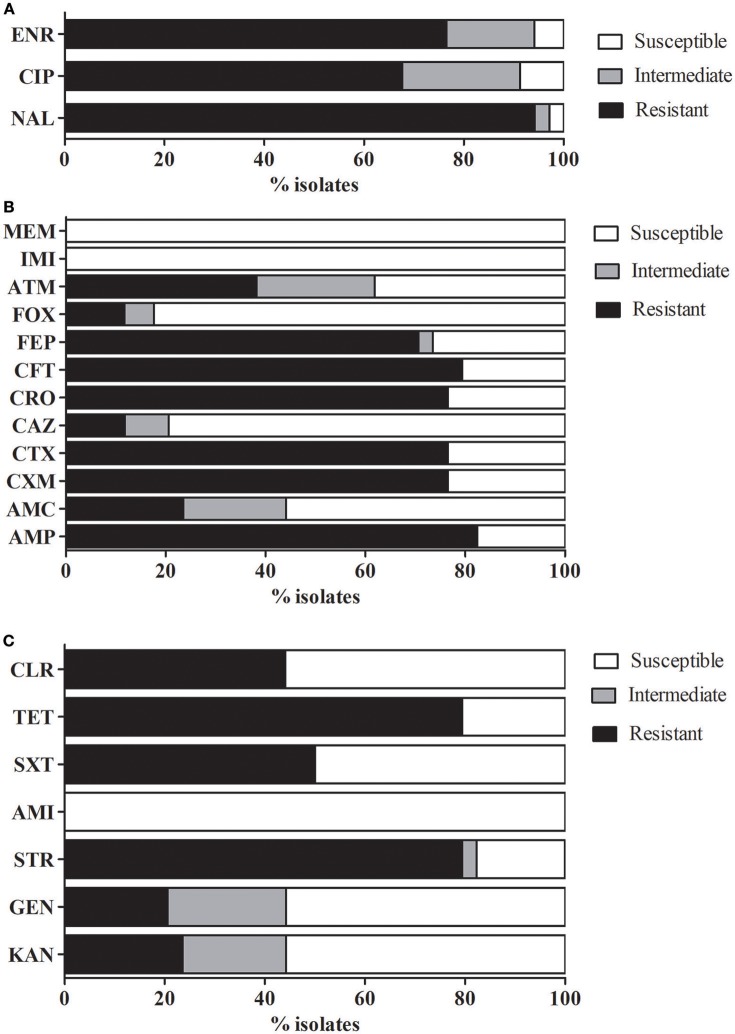
Antimicrobial susceptibility profiles. Percentage of antimicrobial susceptibility in the isolates analyzed. **(A)** Fluoroquinolones, **(B)** β-lactams, and **(C)** Other antimicrobials. AMP, ampicillin; AMC, amoxicillin-clavulanic acid; CXM, cefuroxime; CTX, cefotaxime; CAZ, ceftazidime; CRO, ceftriaxone; CFT, ceftiofur; FEP, cefepime; FOX, cefoxitin; ATM, aztreonam; IMI, imipenem; MEM, meropenem; NAL, nalidixic acid; CIP, ciprofloxacin; ENR, Enrofloxacin; KAN, kanamycin; GEN, gentamicin; STR, streptomycin; AMI, amikacin; SXT, trimethoprim-sulfamethoxazole; TET, tetracycline; and CLR, chloramphenicol.

Further determinations of antimicrobial susceptibility of *mcr-1*-positive strains demonstrated high rates of multidrug resistance, since 85% (29/34) of the tested strains were resistant to at least three different classes of antimicrobial agents (Figure 1).

Simultaneous resistance to colistin and quinolones or fluoroquinolones was relatively high (Figure 1A), since 94% (32/34) of the *mcr-1*-positive strains were resistant to nalidixic acid (NAL), 67.6% (23/34) to ciprofloxacin (CIP), and 76.5% (26/34) to enrofloxacin (ENR). Almost three from every four strains (76.5%) harbored a PMQR marker and the most prevalent determinants were *qnrS* (20/34) and *qnrB* (18/34). Almost three from every four strains (76.5%) harbored a PMQR marker and the most prevalent determinants were *qnrS* (20/34) and *qnrB* (18/34). Other PMQRs such as *qnrA* (2/34), *qnrD* (1/34) and the efflux pumps *oqxAB* (5/34) and *qepA* (5/34) were also identified. These results are consistent with the analysis made by Huang et al. (2009) in isolates from China, who also found a high ratio of *E. coli* strains harboring PMQR determinants and the authors suggest that this fact may be related to the extended use or misuse of antimicrobials in poultry.

Although no significant genotypic relation (*p* > 0.05) was found between *mcr-1*-positive strains and plasmid mediated quinolone resistance genes (PMQR), results obtained in conjugation experiments suggest that fluoroquinolone and colistin resistance can be simultaneously co-transferred, since both transconjugants (EC 190-14 TC and EC 191-07 TC or EC 191-07 TCS) displayed decreased susceptibility to NAL and were positive for *qnrB* gene detection (Table 3). However, the large number of strains carrying genetic determinants for fluoroquinolones in healthy broilers was relatively high; this scenario suggests that other selective forces such as colistin used as AGP (Morales et al., 2012) or therapeutic antimicrobial misuse are driving the selection of fluoroquinolone-resistant bacteria.

#### Resistance to β-lactams and detection of extended spectrum β-lactamase (ESBL) and plasmidic AmpC β-lactamase

The antimicrobial susceptibility analysis showed a relatively high percentage of AMP resistance 82.4% (28/34) among the *mcr-1*-positive strains and a strong relation between susceptibility to both antimicrobials as determined by disk diffusion tests (R: 0.33, *p* < 0.05). Considering the susceptibility showed to AMP, a high percentage of resistance (between 76.5 and 79.4%) was also observed in oxyimino-cephalosporins (CTX, CRO and CFT, a cephalosporin used in veterinary medicine) and FEP (70.6%). However, very little resistance to CAZ and FOX was detected, while all isolates remained susceptible to carbapenems (IMI and MEM) (Figure 1B). In contrast, most clinical *E. coli* strains were found to be susceptible to a wide range of antimicrobials, including carbapenems (Lai et al., 2017).

CTX-M-producing enterobacteria are widespread among human population and an increasing number of reports describes their presence in livestock environments as well as in food from animal origin (Lazarus et al., 2015). Our findings demonstrate that also healthy birds may act as a reservoir of *bla*_CTX−M−2_ and *bla*_CTX−M−14_ genes. In the recent past, CTX-M-2 was the dominant ESBL group among human clinical *Enterobacteriaceae* isolates in South America (Quinteros et al., 2003; Minarini et al., 2007; Saba Villarroel et al., 2017). From 26 extended- spectrum cephalosporins (ESC)-resistant and *mcr-1*-positive strains, 18 strains (18/34, 56%) were CTX-M-2 producers and two produce CTX-M-14. Five strains harbored both CTX-M genes. CMY-2 was identified in 4 strains (3 were also CTX-M-2 producers) (Table 2). According to these results, the main mechanism responsible for TGC resistance was the production of CTX-M cefotaximases which explained the low resistance rates to FOX and CAZ. ESBLs from groups CTX-M-2 and CTX-M-14 were previously identified in *mcr-1*-carrying *E. coli* recovered from human samples (Rapoport et al., 2016) and from wild birds (kelp gulls) in the south of Argentina (Liakopoulos et al., 2016).

The cosmopolitan CTX-M-15 variant belonging to the CTX-M-1 subfamily, which is also widespread in human clinical isolates from Argentina, (Sennati et al., 2012), could not be found in this study. This finding was unexpected since reports from Brazil, where poultry productive systems are similar to Argentina (Botelho et al., 2015), described the presence of the CTX-M-15 ESBL and the coexistence of CTX-M-8 and CMY-2 in *E. coli* isolates recovered from chicken meat.

Many studies in *E. coli* strains, most of them involving isolates from animals, have demonstrated the presence of *mcr-1* gene together with ESBL (Rhouma and Letellier, 2017). In the present work, despite the presence of the CTX-M-2 gene in the parental strain, no co-selection of ESC-resistant was observed in the transconjugants (Table 3).

Although 50% of the *E. coli* strains analyzed carried both sets of ESBL and PMQR genes, no association between the presence of ESBL and a specific PMQR mechanism (*p* > 0.05) was observed. Additionally, *aac (6*′*)-Ib-cr* gene was not detected. It is remarkable the absence of *aac (6*′*)-Ib-cr* gene, and the lack of association between ESBL and PMQR which is usually found in some *Enterobacteriaceae* isolated from human (Andres et al., 2013; Cruz et al., 2013) in Argentina.

A large variability of PMQR determinants was also observed in TGC-sensitive (without ESBL) and *mcr-1*-positive strains with a similar proportion of *qnrB* and *qnrS* genes. To a lesser extent, some of these strains also showed *oqxAB* gene. According to the results of this study, we suggest that *E. coli* strains from broiler chickens could be the reservoir not only of the *mcr-1* gene, but also of PMQR and ESBL genes.

#### Resistance to other antimicrobials-multiple drug resistance (MDR)

Most of the *mcr-1-*positive strains were determined to carry ESBL or PMQR-genes and also most of them were resistant to other classes of antimicrobial agents. This is probably due to the fact that the aforementioned genes are commonly found in mobile elements such as conjugative plasmids that also harbor resistant determinants to different groups of antimicrobials and confer the MDR phenotype. It is of particular concern that 39/41 (95.1%) strains considered in this study (including *mcr-1*-negative strains) expressed a multi-resistance phenotype.

The percentage of strains resistant to aminoglycosides and *mcr-1*-positive strains was variable and drug dependent. Resistance rates to this family was STR>KAN (79.4%, 23.6%) and GEN (20.6%). All strains remained susceptible to AMI (Figure 1C). Resistance to TET (79.4%) was relatively high, as expected considering the extensive use in animal medicine, which is in concordance with previous studies were TET resistance markers are frequently found in *E. coli* strains (Argudín et al., 2017). To a lesser extent, also resistance to SXT (50%) and CLR (44%) was also detected.

## Conclusions

The results highlight that commercial broiler farms can be an important reservoir of *mcr*-1-carrying *E. coli* strains. In fact, the high occurrence of *E. coli* isolates (76%) carrying the *mcr-1* gene is alarming and has not been reported in any other part of the world (Delgado-Blas et al., 2016; Fernandes et al., 2016; Kawanishi et al., 2017; Meinersmann et al., 2017; Monte et al., 2017; Whang et al., 2018). These differences could be associated with the method of screening used in the present report since a higher number of unrelated farms separated from a relatively high distance were considered. Although this may be related to particularities of the productive system, the local practices are quite similar to the ones from other countries in South America that were administering colistin without any restriction. A potential combination of antibiotics used in the productive system, climatic variations and other variables, may influence the spread of *mcr-1* and these scenarios could also contribute to the selection of multi-resistant bacteria.

In this study, we determined the presence of resistance determinants in colistin-resistant *E. coli* strains from the environment of an intensive production system such as broiler chickens destined to consumption. A wide range of phenotypic resistance to both antibiotics in veterinary and human medicine was identified and resistance to colistin, quinolones and β-lactams was observed in the analyzed strains. The proportion of resistance to other antimicrobial families (SXT, TET, CLR, and aminoglycoside) was relatively high, underlining the presence of a large number of isolates with a MDR profile. The ability the co-transference of the *qnrB* determinant on the *mcr-1*-positive transconjugants was corroborated, which suggests that these resistance genes are likely to be located in the same plasmid thus transforming it into a more successful clone.

## Author contributions

JD, LR, GG, JC, and MF participated in the design of the study. JD, RF, and DC performed the experiments. JD, LR, JC, and MF analyzed the data. JD, LR, PC, and MF collected *E. coli* strains. JD, LR, PC, JC, and MF wrote the paper. All authors contributed to the critical revision of the manuscript and have seen and approved the final draft. All authors read and approved the final manuscript.

### Conflict of interest statement

The authors declare that the research was conducted in the absence of any commercial or financial relationships that could be construed as a potential conflict of interest.

## Footnotes

^1^http://www.eucast.org

## Supplementary material

Figure S1 Phylogenetic tree and patterns of ERIC-PCR on agarose gel electrophoresis.
