# Supplementary figures and images for: Simultaneous Carriage of mcr-1 and Other Antimicrobial Resistance Determinants in Escherichia coli From Poultry
Source: Front Microbiol. 2018 Jul 25;9:1679. doi: 10.3389/fmicb.2018.01679 (PMC6068390; doi:10.3389/fmicb.2018.01679)

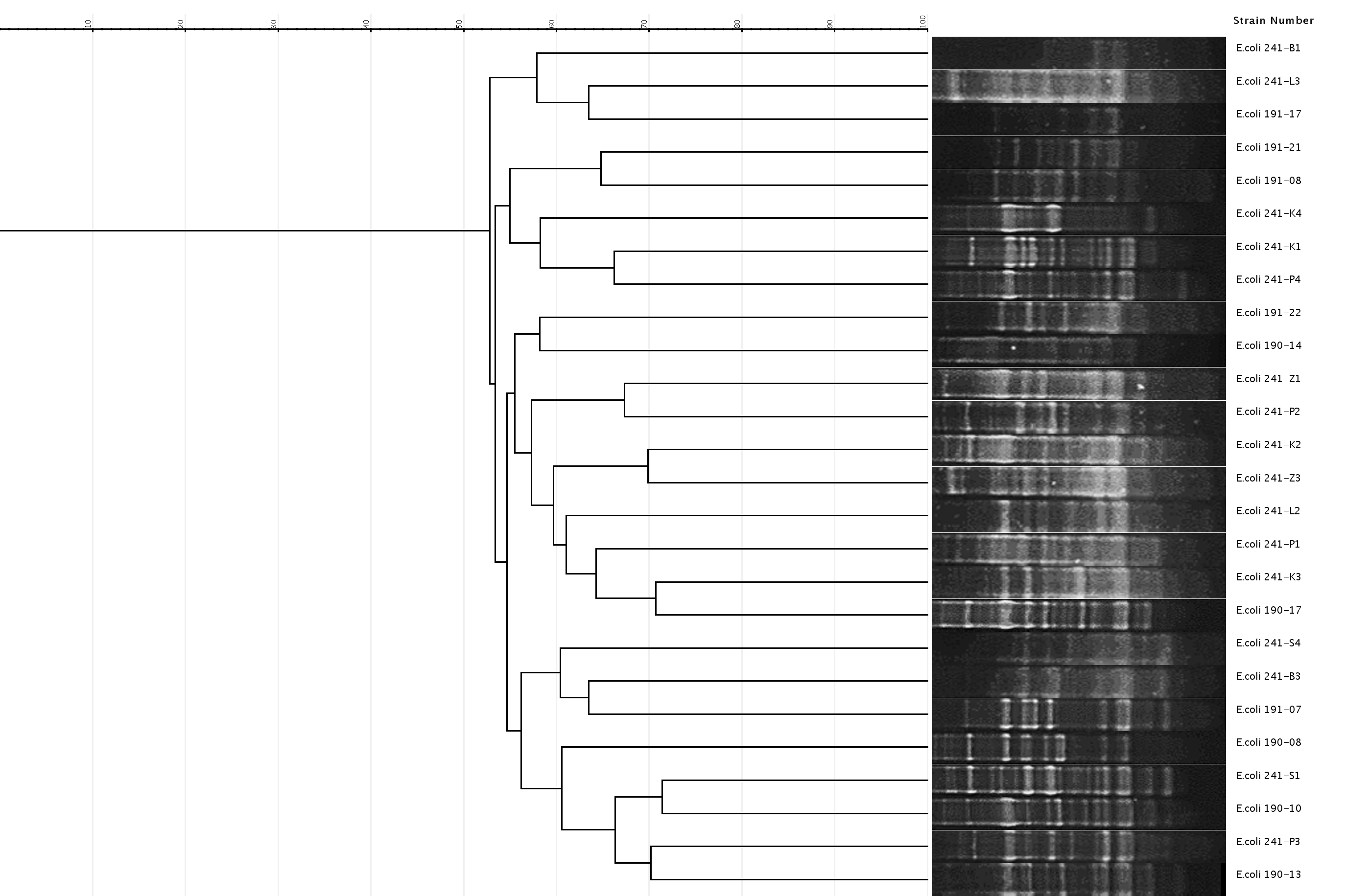

Supplement: Figure S1 — Phylogenetic tree and patterns of ERIC-PCR on agarose gel electrophoresis. [file Image_1.tif]
